# Mindfulness-based intervention and sexuality: a systematic review

**DOI:** 10.47626/2237-6089-2021-0459

**Published:** 2024-11-06

**Authors:** Amaia Miren Ciaurriz Larraz, Alejandro Villena Moya, Carlos Chiclana Actis

**Affiliations:** 1 Unidad Sexología Clínica Madrid Spain Unidad Sexología Clínica, Consulta Dr. Carlos Chiclana, Madrid, Spain.

**Keywords:** Sexual disorders, aware, treatments, evidence, sexual dysfunctions

## Abstract

**Objectives:**

Mindfulness has generated considerable interest in the last 2 decades in clinical and research settings. The efficacy of mindfulness has been evaluated for the sexual dysfunctions recognized by the Diagnostic and Statistical Manual of Mental Disorders, 5th edition (DSM-5) and other sexual problems, such as compulsive sexual behavior disorder (CSBD), also known as sex addiction or hypersexuality. Here, we review the evidence for various mindfulness-based treatments (MBT), such as mindfulness-based cognitive-behavioral treatment or mindfulness-based relapse prevention, for different problems related to sexuality to answer our question: "Are mindfulness-based treatments effective in reducing the symptomatology of sexuality-related disorders?"

**Methods:**

Through a systematic search conducted following the Preferred Reporting Items for Systematic reviews and Meta-Analyses (PRISMA) guidelines, we found 11 studies that met the following inclusion criteria: (I) articles using MBT for sexuality-related problems; (II) clinical population; (III) no date range limits were applied; (IV) only empirical studies were included; (V) language; and (VI) quality of studies.

**Results:**

Evidence shows that mindfulness practice could be effective for some sexual disorders, such as female sexual arousal/desire disorder. However, due to scarcity of studies on other sexual problems such as situational erectile dysfunction, genitopelvic pain/penetration disorder, childhood sexual abuse, or CSBD, the findings cannot be generalized.

**Conclusion:**

There is evidence that mindfulness-based therapies can reduce the symptomatology associated with various sexual problems. However, more studies are needed for these sexual problems. The study concludes with a discussion of future directions and implications.

## Introduction

### Conceptualization of mindfulness

In the last 2 decades, mindfulness has generated considerable interest in clinical and research settings,^1–5^ It has been defined as "the ability to pay attention in a particular way, in the present moment to the body and mind, with purpose and without judgment."^6^ From a scientific perspective, mindfulness has been described as a mental function that allows us to keep the focus of our attention on an immediate experience of the present.^7^ According to Miró,^8^ mindfulness implies "being free of worries and anticipations. It requires attention to what is happening to us and what is happening, to look and formulate the intention to see."

Mindfulness works through four main mechanisms: attentional regulation, changes in the perspective of oneself, emotional regulation, and body awareness.^9^ These mechanisms have been used in a wide range of psychiatric disorders, such as depression, stress, insomnia, anxiety, and binge eating disorder, with promising results.^10–14^

### Mindfulness and sexual difficulties

Sexual dysfunctions are a heterogeneous group of disorders that are characterized by a clinically significant disturbance in a person's ability to respond sexually or to experience sexual pleasure.^15^ The Diagnostic and Statistical Manual of Mental Disorders, 5th edition (DSM-5), covers the following sexual dysfunctions: delayed ejaculation, erectile disorder, female orgasmic disorder, female sexual interest/arousal disorder, genitopelvic pain/penetration disorder, male hypoactive sexual desire disorder, and premature (early) ejaculation.^16^ However, the categorization of both male and female sexual dysfunctions underwent several modifications in the transition from DSM-IV-TR^15^ to DSM-5.^16^ First, two of the disorders, named "female hypoactive desire disorder" and "female arousal disorder" in the DSM-IV-TR,^15^ were lumped into a single disorder in the DSM-5: "female sexual interest/arousal disorder."^16^ Another change was to include dyspareunia, vaginismus, and male sexual pain.^15^ in one disorder called "genitopelvic pain/penetration disorder."^16^ In addition, a small change was to eliminate the word "male" in the erectile disorder.^16^ Lastly, the "male orgasmic disorder"^15^ was modified with "delayed ejaculation."^16^

The possible effectiveness of mindfulness has been evaluated for the following sexual dysfunctions: female sexual arousal/desire disorder, genitopelvic pain/penetration disorder, and erectile dysfunction (ED). Female sexual arousal/desire disorder is mainly displayed by a reduced interest in sexual activity or by an absence of sexual arousal or pleasure.^16^ This disorder can be manifest in one of the two following ways: (a) decreased genital sexual response in the absence of genital awareness (physiological) or (b) decreased sexual affect (subjective sexual arousal) with a negative mental engagement during sexual activity.^16^ Genitopelvic pain/penetration disorder can be presented in a number of ways.^16^ One of these is known as vestibulodynia (vulvar vestibulitis), which is an increased sensitivity to pain at the vaginal opening (vestibule) to the point that even light touch or stimulation is painful.^17^ To be more specific, provoked vestibulodynia (PVD) is the term used to describe superficial pain confined to the vulvar vestibule, provoked by touch.^18^ ED refers to the persistent inability to achieve or maintain an erection or to a reduction in erection rigidity.^16^ Situational ED therefore, occurs when that disability is due to the situation, couples, or certain types of stimulation, rather than being a generalized disability.^16^

In addition to the use of mindfulness to address the sexual dysfunctions recognized by the DSM-5,^16^ the efficacy of mindfulness has also been evaluated for other sexual problems, such as compulsive sexual behavior disorder (CSBD), also known as sex addiction or hypersexuality. With the arrival of the 11th edition of the International Classification of Diseases (ICD-11),^19^ CSBD was approved as a specific category within impulse control disorder. The defining criteria proposed are the following: (a) repetitive sexual behaviors that become the main focus of the person's life; (b) numerous unsuccessful efforts to control or significantly reduce one's sexual behavior; (c) continuing to engage in sexual conduct despite the adverse consequences; and (d) continuing with sexual behavior even when pleasure is not derived from it or is very little.^19^

Finally, in the scientific literature, it is already known that both biological and psychological factors are related to sexual dysfunctions. Childhood sexual abuse (CSA) has been identified as a precipitating factor for impaired sexual functioning in adulthood. Although sexual difficulties related to a history of CSA are common, studies that have evaluated effective treatments addressing sexual distress are scarce.^20^

Distressing sexual interactions may produce negative thoughts and judgments.^21^ Traditionally, treatments in the field of sexual medicine have tried to increase the connection with the body. Mindfulness complies with these elements, producing changes in the perspective of oneself and body awareness.^9^ Therefore, mindfulness may be an effective way of re-routing one's focus away from negative memories or anticipated sexual problems and onto the sensations that are unfolding in the moment.^21^ For this reason, mindfulness has been incorporated into sexual medicine in recent years.^22^ Based on the first promising results of use of mindfulness in clinical sexology, it seems interesting to review the efficacy of mindfulness-based treatment (MBT) in sexual medicine.

Therefore, the present systematic review was conducted following the Preferred Reporting Items for Systematic reviews and Meta-Analyses (PRISMA) guidelines with the primary aim of assessing the efficacy of MBT for sexual dysfunctions (hypoactive sexual desire, sexual arousal disorders, sexual pain disorders, ED) and other sexual problems (sexual abuse and/or CSBD) in a clinical population comprising both men and women. Our review question was: "Are MBT effective in reducing the symptomatology of sexuality-related disorders?"

## Material and methods

### Information sources and search strategy

The search was undertaken using the Biblioteca de Universidad de Navarra (UNIKA) metasearch engine. This metasearch includes the multidisciplinary databases Scopus, Web of Science, and Dialnet and also the specialized bibliographic databases PubMed and PsycINFO.

The search terms used in the present systematic review were "Mindfulness" AND ("Sexuality" OR "Sexual dysfunctions" OR "Sex Therapy" OR "Sexual Problems" Or "Sexology" OR "Sexual Difficulties").

### Eligibility criteria

This systematic review followed the following eligibility criteria: (I) articles using MBT for sexuality-related problems (sexual dysfunctions included in the DSM-5: hypoactive sexual desire, sexual arousal disorders and orgasmic disorders, CSBD included in ICD-11, and other sex-related problems such as sexual abuse); (II) clinical population: adult (> 18 years old) men and women with sexual problems; (III) no date range limits were applied. The most recent search was conducted on January 8, 2023; (IV) only empirical studies were included; (V) languages: English and Spanish; and (VI) strong or moderate quality, measured by the Quality Assessment Tool for Quantitative Studies.^23^

### Data collection process

A two-step process was used to assess the results of the literature search. First, two reviewers (AMC and AV) screened all potential articles individually using title and abstracts prior to retrieval of full text. For the second level of the screening, articles identified for full review were further screened according to the eligibility criteria. Differences of opinion between the two reviewers were resolved through consensus.

### Data items

We extracted article data including the full reference, main aims, sample characteristics and sample size, description of methodology, study design, and results.

## Results and discussion

### Study selection

**Figure 1:**
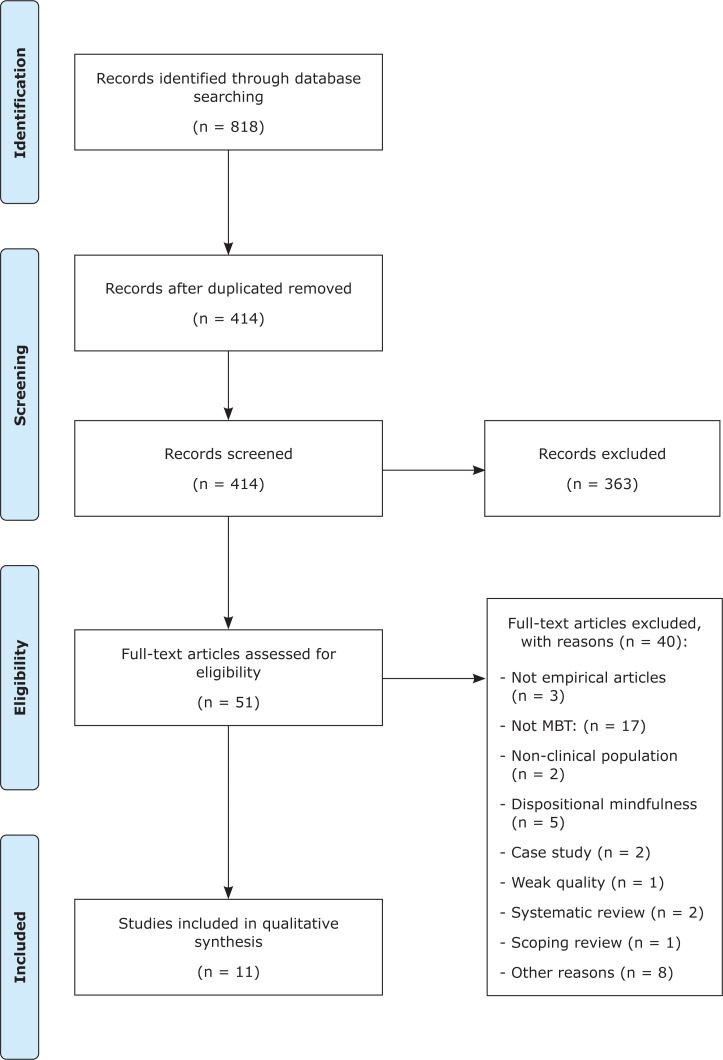
Preferred Reporting Items for Systematic reviews and Meta-Analyses (PRISMA) flow diagram

A total of 818 records were retrieved from our literature search. After removing 404 duplicates, 363 of the 414 remaining articles were excluded by screening titles and abstracts. The remaining 51 articles were screened at the full-text level. Eleven of the 51 full-text articles screened were ultimately included in the present review (Figure 1).

### Study characteristics

Table 1 lists the main characteristics of the studies (first author and year of publication, study design, sample and sample size [n], purpose of the study, procedure, measures, and results).

**Table 1:** Summary of included articles

First author	Study design	Study quality	Sample	Sample size (n)	Study purpose	Procedure	Measures	Results
Brotto^24^	Quantitative, cross-sectional	Strong	Women with sexual arousal disorder	22	To analyze the effectiveness of a brief psychoeducational intervention in women with sexual arousal disorder	Three sessions of 1 h combined elements of cognitive and behavioral therapy with education and mindfulness training	DASA, FSFI, FSDS, DAS, BDI, SF-36, Film Scale, VPA	Significant increase: desire, arousal, orgasm, and satisfaction subscales of the FSFI (as well as total score), mental sexual excitement and genital tingling/throbbing measured using the FSFI. Sexual distress significantly decreased (FSDS). Women who were initially more depressed showed a more marked improvement in pleasant sexual genital sensations (DASA).
Brotto^25^	Quantitative, cross-sectional	Strong	Women with sexual desire/interest and/or sexual arousal disorders related to cancer	26	To study the effectiveness of mindfulness in women with sexual desire/interest disorder and/or sexual arousal disorders not related to cancer	90-minute sessions, spaced 2 weeks apart	FSFI, FSDS, SIDI, DASA, DAS, BDI, the film scale	Significant increase in desire (measured by FSFI, SIDI, and FSDS) and in genital wetness (subscale of DASA)
Brotto^26^	Quantitative, cross-sectional	Strong	Women with a history of childhood sexual abuse and sexual distress	20 (n = 8 CBT vs. n = 12 MBT)	To compare brief interventions using CBT or MBT in women with sexual difficulties and distress related to a history of abuse	20 women were randomized to two sessions of either CBT or MBT.	FSDS, FSFI, Assessment of Child Sexual Abuse History, VPA	Significant decrease in sexual distress in both groups. The MBT group significantly increased concordance between genital and subjective sexual arousal by increasing the amount of subjective sexual arousal compared to CBT group and pre-treatment
Brotto^27^	Quantitative, longitudinal	Moderate	Women with distressing sexual desire and/or sexual arousal problems	31	To evaluate a mindfulness-based cognitive behavioral intervention for sexual dysfunction in gynecologic cancer survivors vs. waitlist	Three 90-minute MB-CBT sessions vs. 2 months of a wait-list control	FSFI, FSDS, BDI, VPA, SFQ	Significant improvements in desire, arousal, lubrication, orgasm, satisfaction, and FSFI total scores. Sexual distress decreased (FSDS).
Brotto^28^	Quantitative, longitudinal	Strong	Women with sexual desire problems	Immediate treatment (n = 68) vs. delayed treatment (n = 49)	To study the effectiveness of MBT in women seeking treatment for low sexual desire and arousal	Four 90-min group MBCST sessions	SIDI, FSDS, FSFI, DASA, BDI, DAS, FFMQ	The treatment group significantly improved sexual desire, sexual arousal, lubrication, sexual satisfaction, and overall sexual functioning (vs. control group). Sex-related distress, orgasmic difficulties and depressive symptoms significantly decreased in both conditions.
Brotto^29^	Quantitative, longitudinal	Moderate	Women with PVD	Treatment (n = 62) vs. wait-list (n = 23)	To evaluate mindfulness-based therapy as a treatment for PVD	Four-session group treatment ("improved") that relied on mindfulness meditation skills with education and cognitive theory	Pain-related endpoints, changes in allodynia, PISES, PCS, PVAQ, FSDS, FSFI, BDI, FFMQ, STAI	Significant beneficial effects of a brief mindfulness-based group intervention for women with PVD on both cotton swab-induced vestibular pain and psychological measures of pain.
Brotto^30^	Quantitative, cross-sectional	Strong	Women with sexual arousal/desire difficulties	79	To examine the effects of mindfulness-based sex therapy on sexual arousal concordance in a sample of women with sexual desire/arousal difficulties	Mindfulness-based sex therapy integrating psychoeducation, sex therapy, and mindfulness-based skills.	Assessment of Psychophysiological and Subjective Sexual Arousal, Discrete Measure of Sexual Response and Affect,* FSFI, SIDI, FSDS	Genital-subjective sexual arousal concordance significantly increased from pre-treatment levels, with changes in subjective sexual arousal predicting contemporaneous genital sexual arousal (but not the reverse).
Paterson^31^	Quantitative, longitudinal	Strong	Women with a diagnosis of sexual interest/arousal disorder	26	To evaluate the efficacy of MBCT-S	8 sessions of group MBCT-S	SIDI, FSDS-R, FSFI, FFMQ, BDI-II, SCS, MAIA, RRS, ASI-3	Compared to baseline, women reported significant improvements in sexual desire, overall sexual function, and sex-related distress, regardless of treatment expectations, relationship duration, and low desire duration. Depressed mood and mindfulness also significantly improved and mediated increases in sexual function.
Bossio^32^	Quantitative, longitudinal	Moderate	Men with situational ED	10	To implement an adapted empirically supported treatment protocol for female sexual dysfunction for men with situational ED	4-week mindfulness-based treatment group included daily home-practice activities, psychoeducation, sex therapy, and mindfulness skills.	IIEF, Relationship Assessment Scale, FFMQ	Comparisons between Time 1 (prior to treatment) and Time 3 (6 months after treatment) self-reports suggested that this treatment protocol holds promise impacting erectile functioning, overall sexual satisfaction, and non-judgmental observation of one's experience.
Holas^33^	Quantitative, cross-sectional	Moderate	Men with a diagnosis of CSBD	13	Examine if an MBRP can lead to clinical improvement in CSBD	An 8-week MBRP intervention that included guided meditation, experiential exercises, inquiry, psychoeducation, and discussion	BPS, HADS, OCI-R	After the mindfulness intervention, participants spent significantly less time engaging in problematic pornography use. MBRP also resulted in a reduction of the symptoms of problematic pornography use, in emotional distress and reduced depressive symptoms and decreased obsessive-compulsive symptoms. Did not find a decrease in time spent in masturbation or dyadic sex.
Rashedi^34^	Quantitative, longitudinal Randomized parallel-group clinical trial	Strong	Women with a diagnosis of sexual desire disorder	70	To determine the effect of MBCST on improving sexual desire, distress, self-disclosure, and function in women with sexual desire disorder	Four 90-120 minute long weekly group sessions. Questionnaires were completed immediately, 4 and 12- weeks after the interventions.	HSID, FSDS-R, FSFI, SSDQ	The treatment group (vs. control group) significantly improved sexual desire, sexual distress, self-disclosure, and sexual function during and after (4 and 12 weeks) the intervention.

ASI-3 = Anxiety Sensitivity Index-3; BDI = Beck Depression Inventory; BPS = Brief Pornography Screener; CBT = cognitive-behavioral treatment; CSBD = compulsive sexual behavior disorder; DAS = Dyalic Adjustment Scale; DASA = Detailed Assessment of Sexual Arousal; ED = erectile dysfunction; FFMQ = Five Facet Mindfulness Questionnaire; FSDS = Female Sexual Distress Scale; FSFI = Female Sexual Function Index; HADS = Hospital Anxiety and Depression Scale; HSID = Halbert Index of Sexual Desire; IIEF = International Index of Erectile Functioning; MAIA = Multidimensional Assessment of Interoceptive Awareness; MB-CBT = mindfulness-based cognitive-behavioral treatment; MBCST = mindfulness-based cognitive-behavioral sex therapy; MBCT-S = mindfulness-based cognitive therapy for sexuality; MBRP = mindfulness-based relapse prevention; MBT = mindfulness-based treatment; OCI-R = Obsessive-Compulsive Inventory-Revised; PCS = Pain Catastrophizing Scale; PISES = Painful Intercourse Self-Efficacy Scale; PVAQ = Pain Vigilance and Awareness Questionnaire; PVD = Provoked Vestibulodynia; RRS = Ruminative Responses Scale; SCS = Self-Compassion Scale; SF-36 = SF-36 Quality of Life Questionnaire; SFQ = Sexual Function Questionnaire (the Treatment Impact and Relationship subscales); SIDI = Sexual Interest and Desire Inventory; SSDQ = Sexual Self-Disclosure Questionnaire; STAI = State-Trait Anxiety Inventory; VPA = psychophysiological recording by vaginal pulse amplitude.

* A 33-item self-report questionnaire was used to assess subjective arousal and affective reactions to erotic films.

### Sexual arousal disorder and sexual desire disorder

Most of the articles included in this systematic review explored the effectiveness of mindfulness in sexual arousal or sexual desire disorder in women. These articles suggest that mindfulness exercises or MBT reduce the symptomatology of these disorders. However, they assessed effectiveness using different psychometric instruments.

Regarding sexual arousal disorder, two types of samples were included: women with sexual arousal disorder and women with sexual arousal disorder following a gynecologic cancer. Mindfulness was effective to improve sexual arousal in these women. In addition, it has been suggested that a psychoeducational intervention including CBT with psychoeducation and mindfulness training may improve subjective sexual arousal.^24^ Brotto et al.^30^ used a mindfulness-based sex therapy which integrated psychoeducation, sex therapy, and mindfulness-based skills, and they found that the increase of genital sexual arousal is indirectly related to the increase in subjective sexual arousal after the mindfulness intervention. Therefore, if there is a prior subjective sexual arousal, genital arousal may increase. According to these results, it can be said that mindfulness practice can help increase sexual arousal in women not only directly (increasing genital arousal), but also indirectly (increasing subjective or mental arousal and, consequently, genital arousal).

With regard to sexual desire, significant improvements in sexual desire were described for women survivors of gynecological cancer and for women with low sexual desire in comparison with baseline and with a healthy control group. This finding has been observed in relation to different types of treatment, such as mindfulness-based cognitive-behavioral treatment (MB-CBT) and mindfulness-based cognitive therapy for sexuality.^27,28,31^ It was also seen that sexual desire, sexual distress, and self-disclosure significantly improved in women with low sexual desire after a mindfulness-based cognitive-behavioral sex therapy (MBCST).^34^ The sexual function domains (including sexual arousal, lubrication, orgasm, satisfaction) also improved in the intervention group (vs. control group).^34^

### PVD

Brotto et al.^29^ analyzed a four-session mindfulness-based group treatment for women suffering from PVD. The treatment relied on mindfulness meditation skills with education and cognitive theory. After the brief mindfulness-based group intervention, a significant beneficial effect on vestibular pain and psychological measures of pain was found for women with PVD. Therefore, the MBT not only helped to reduce the sensation of physical pain, but also was effective for reducing subjective pain.

### Situational ED

There is no specific MBT for ED. Therefore, Bossio et al.^32^ implemented an adapted, empirically supported treatment protocol for female sexual dysfunction with men with situational ED. This four-session group treatment integrated elements of mindfulness, sex therapy, and psychoeducation. Comparisons between pretreatment and 6-month posttreatment self-reports suggested that this protocol is promising with regard to erectile function, general sexual satisfaction, and non-judgmental observation of one's own experience.

### CSBD

There are few studies that talk about the application of mindfulness in patients with CSBD; two studies talk about the relationship between dispositional mindfulness and hypersexuality, showing that they are inversely and negatively related^35,36^ and another presents a case study indicating improvements in symptomatology.^37^ On the other hand, no empirical studies have been conducted to analyze the effectiveness of a treatment based on mindfulness for patients with CSBD. The study included in this review is a pilot study that examined whether MBRP can lead to clinical improvement in 12 males with CSBD.^33^ The results indicate that after the intervention, participants spent significantly less time engaging in problematic pornography use. The intervention also reduced the symptoms of problematic pornography use, emotional distress, depressive symptoms, and obsessive-compulsive symptoms. However, there was no decrease in time spent in masturbation or dyadic sex. This first study examining MBT in the context of CSBD presents promising preliminary results. Nonetheless, more studies are needed with larger and statistically more powerful samples to yield more reliable and generalizable results.

### Sexual abuse

As has been seen throughout this review, there is growing evidence about the benefits of mindfulness in treating sexual difficulties. However, no randomized controlled studies have been done in populations with a history of sexual abuse. A pilot study with women who suffered CSA compared two sessions of MBT (n = 12) with CBT (n = 8), finding that MBT proved to be significantly more effective than CBT in improving the concordance between genital sexual arousal and subjective sexual arousal.^26^

An analysis based on hierarchical linear modeling to assess changes in concordance between subjective and genital sexual arousal showed that women in the MBT group experienced a significantly higher subjective sexual arousal response compared to the CBT group and to before treatment.^26^ Additionally, both groups experienced a significant decrease in sexual distress.^26^ Therefore, this pilot study supports the further study of mindfulness-based approaches in the treatment of sexual difficulties related to history of CSA and characterized by a disconnect between genital and subjective sexual response.

### Limitations and future studies

This study has some limitations that should be born in mind. First, most of the studies included focus on women with sexual problems who have sought treatment. Consequently, the findings are not generalizable to women who have not sought treatment. Second, there is limited literature on MBT in men, for which reason future studies could focus on men. Finally, further studies are required for each of the sexual problems included in this review to enable firm conclusions to be drawn about the efficacy of MBT. However, while a limitation, it could also be a strong point for this study that almost all the studies are from the same group, allowing firm conclusions to be drawn for this group.

## Conclusion

The present systematic review provides evidence on the efficacy of MBTs to reduce the symptomatology associated with various sexual problems such as sexual arousal disorder and/or sexual desire disorder, PVD, and sexual abuse in women, or situational ED and hypersexuality in men. However, more studies are needed in this line to obtain solid conclusions in this respect.
